# Functional Spectroscopy Mapping of Pain Processing Cortical Areas During Non-painful Peripheral Electrical Stimulation of the Accessory Spinal Nerve

**DOI:** 10.3389/fnhum.2019.00200

**Published:** 2019-06-13

**Authors:** Janete Shatkoski Bandeira, Luciana da Conceição Antunes, Matheus Dorigatti Soldatelli, João Ricardo Sato, Felipe Fregni, Wolnei Caumo

**Affiliations:** ^1^Laboratory of Pain and Neuromodulation, Universidade Federal do Rio Grande do Sul (UFRGS), Porto Alegre, Brazil; ^2^Department of Nutrition, Health Science Center, Universidade Federal de Santa Catarina (UFSC), Florianópolis, Brazil; ^3^Department of Mathematics and Statistics, Universidade Federal do ABC, Santo André, Brazil; ^4^Physical Medicine & Rehabilitation, Berenson-Allen Center for Noninvasive Brain Stimulation, Department of Neurology, Beth Israel Deaconess Medical Center, Harvard Medical School, Boston, MA, United States; ^5^Laboratory of Pain and Neuromodulation, Department of Pain and Anesthesia in Surgery, Hospital de Clínicas de Porto Alegre, Universidade Federal do Rio Grande do Sul (UFRGS), Porto Alegre, Brazil

**Keywords:** cortical activation, near infrared spectroscopy, peripheral nerve stimulation, electrical nerve stimulation, electroacupuncture, accessory spinal nerve

## Abstract

Peripheral electrical stimulation (PES), which encompasses several techniques with heterogeneous physiological responses, has shown in some cases remarkable outcomes for pain treatment and clinical rehabilitation. However, results are still mixed, mainly because there is a lack of understanding regarding its neural mechanisms of action. In this study, we aimed to assess its effects by measuring cortical activation as indexed by functional near infrared spectroscopy (fNIRS). fNIRS is a functional optical imaging method to evaluate hemodynamic changes in oxygenated (HbO) and de-oxygenated (HbR) blood hemoglobin concentrations in cortical capillary networks that can be related to cortical activity. We hypothesized that non-painful PES of accessory spinal nerve (ASN) can promote cortical activation of sensorimotor cortex (SMC) and dorsolateral prefrontal cortex (DLPFC) pain processing cortical areas. Fifteen healthy volunteers received both active and sham ASN electrical stimulation in a crossover study. The hemodynamic cortical response to unilateral right ASN burst electrical stimulation with 10 Hz was measured by a 40-channel fNIRS system. The effect of ASN electrical stimulation over HbO concentration in cortical areas of interest (CAI) was observed through the activation of right-DLPFC (*p* = 0.025) and left-SMC (*p* = 0.042) in the active group but not in sham group. Regarding left-DLPFC (*p* = 0.610) and right-SMC (*p* = 0.174) there was no statistical difference between groups. As in non-invasive brain stimulation (NIBS) top-down modulation, bottom-up electrical stimulation to the ASN seems to activate the same critical cortical areas on pain pathways related to sensory-discriminative and affective-motivational pain dimensions. These results provide additional mechanistic evidence to develop and optimize the use of peripheral nerve electrical stimulation as a neuromodulatory tool (NCT 03295370— www.clinicaltrials.gov).

## Introduction

Pain processing physiology involves inter-related individual systems, with discriminative, affective, cognitive and social domains, leading to a magnitude of physical and emotional expressions (Melzack, 2001; Chapman et al., 2008). Advances in neuroscience attempted to map brain areas and pathways involved in this neural network, bringing a better understanding of structural and functional brain connectivity. The prefrontal cortex (PFC) has been increasingly associated with pain processing because of its interconnections, including efferent signals to periaqueductal gray (PAG) and dorsal horn neurons (Ong et al., 2019). As an associative cortex, the dorsolateral prefrontal cortex (DLPFC) mediates appraisal to a rewarding stimulus, regulation of emotion and behavior and “keeping pain out of mind” function, that is, moving attention to other things rather than nociception (Wiech et al., 2008). DLPFC is also related to depression and emotional pain aspects related to anxiety (O’Connell et al., 2010). Still, musculoskeletal and neuropathic pain are strongly correlated to motor cortex (MC) and its connections and has been related to pain and cognitive dysfunction by cortico-striatal-thalamo-cortical loops (CSTC; Leite et al., 2017). Afferent nociceptive information that crosses mediodorsal thalamus and anterior cingulate cortex (ACC) reaches DLPFC, which is related to affective-motivational aspects of pain. In turn, the sensory-discriminative dimension of pain involves spinothalamic tract pathway to ventrobasal lateral thalamus and then to sensorimotor cortex (SMC), which in turn anatomically and functionally involves MC, premotor cortex (PMC), supplementary motor area (SMA) and primary somatosensory cortex (S1; Ohara et al., 2005; Hadjipavlou et al., 2006; Yaksh and Luo, 2007). The importance to study the cortical processing of pain in these two target areas, nominally DLPFC and SMC, is to extend data upon the therapeutic approaches effects at the cortical level.

Peripheral electrical stimulation (PES) is being used as a non-pharmacological tool for clinical rehabilitation and treatment of pain presumably by an upward effect inducing reorganization of segmental and central networks (bottom-up outcomes; Chipchase et al., 2011a; Rossini et al., 2015; Chakravarthy et al., 2016). The postulated mechanisms include modulation of the descending modulatory system, release of peptides and endorphins at central and peripheral levels, improvement in motor recruitment, local anti-inflammatory effects, regulation of autonomic activity and changes in long-term depression (LTD)/long-term potentiation (LTP) at synaptic sites (Sandkühler, 2000; Jiang et al., 2013; Zhang et al., 2014). Neurophysiological and neuroimaging studies with PES has shown cortical hemodynamic outcomes in contralateral somatosensory cortex (SSC) and SMC to painful/non-painful type of stimulus, dependent on intensity, in the upper body (median nerve, hand or head) towards activation, using functional near infrared spectroscopy (fNIRS) devices (Tanosaki et al., 2001, 2003; Franceschini et al., 2003; Niederhauser et al., 2008; Takeuchi et al., 2009; Hu et al., 2014; Muthalib et al., 2018) and functional magnetic resonance imaging (fMRI; Blickenstorfer et al., 2009). Lee et al. (2013) correlated the changes in the amplitude of the oxygenated and de-oxygenated hemoglobin with fNIRS with the pain scores on the visual analog scale (VAS) reported by volunteers after applying pain stimulus to the right thumb. Using fNIRS, neuromuscular electrical stimulation (NMES) above motor threshold with evoked pain activated contralateral SMC and bilateral PFC (Muthalib et al., 2015). Aasted et al. (2016) found deactivation of frontal lobe with fNIRS after applying a painful stimulus. Subsequent studies have found different patterns of activation/deactivation comparing painful to non-painful and even paresthetic stimuli using diffuse optical tomography (Becerra et al., 2008, 2009) and fNIRS (Yücel et al., 2015).

Different PES techniques are being studied to improve understanding the mechanism of action and potential indications to pain treatment. Electroacupuncture (EA) can help to treat chronic neck pain (Seo et al., 2017), chronic back pain (Lam et al., 2013) and fibromyalgia (Salazar et al., 2017). Intramuscular electrical stimulation (IMS) with needles improved pain and disability in patients with osteoarthritis (de Graca-Tarragó et al., 2016) and chronic miofascial pain (Couto et al., 2014; Botelho et al., 2018). In previous studies using transcranial magnetic stimulation (TMS), IMS reduced the excitability of the cortical spinal pathway, decreased motor evoked potential (MEP) and intracortical facilitation (ICF) and increased current silent period (CSP; Botelho et al., 2016; Tarragó et al., 2016). NMES studies have demonstrated peripheral neuromuscular adaptations such as increased muscle strength and metabolism, as well as spinal and supraspinal responses (Blickenstorfer et al., 2009; Chipchase et al., 2011a,b; Muthalib et al., 2015). PES can also generate afferent signals for nerve-machine interfaces, that can be used in amputated members rehabilitation, for example (Tan et al., 2015; Ghafoor et al., 2017). Complementary, top-down techniques such as non-invasive brain stimulation (NIBS) are being strongly studied to successfully treat chronic pain by the application of an electrical field on central neural tissue (Castillo Saavedra et al., 2014; Jensen et al., 2014).

Likewise, there is consistent evidence upon vagus nerve (VN) stimulation with an implantable device to aim epilepsy treatment, including potential to help to treat some neuropsychiatric conditions (Hachem et al., 2018). Using fMRI, VN transcutaneous stimulation *via* cervical and auricular sites demonstrated widespread activity in the nucleus of the solitary tract, spinal trigeminal nucleus (TN), locus coeruleus and cortical areas (Frangos et al., 2015; Yakunina et al., 2017; Frangos and Komisaruk, 2017). Still, occipital and trigeminal nerve are being studied and seem to have a role on pain autonomic response and headache treatment (Rigo et al., 2014; Chassot et al., 2015; Chou et al., 2017; Waki et al., 2017). Another peripheral nerve with a close connection with the VN is the accessory spinal nerve (ASN). It is the eleventh cranial nerve formed by a spinal portion from C1 to C4, and a cranial portion from nucleus ambiguous, which also forms VN (Sarrazin et al., 2013; Liu et al., 2014; Shoja et al., 2014). At the level of jugular foramen, the ASN is connected to VN *via* internal ramus or *pars vagalis*. The ASN has a superficial landmark in the posterior cervical triangle and innervates the sternocleidomastoid and trapezius muscles where it receives sensory, proprioceptive and autonomic fibers *via* vagal anastomoses (Benninger and McNeil, 2010; Mitsuoka et al., 2017). In this way, ASN can be an interesting target for its anatomical characteristics and technical facility, accessible to needles and electrodes, regarding new targets for non-invasive therapeutic interventions.

To assess cortical activation, we choose Functional Near Infrared Spectroscopy (fNIRS). It is a non-invasive neuroimaging method used to evaluate cortical function by calculating relative concentrations of oxygenated hemoglobin (HbO), de-oxygenated hemoglobin (HbR) and total hemoglobin (Total-Hb) in cortical capillary networks. Brain activity produces increased oxygen consumption, which is accompanied by increased cerebral blood flow due to neurovascular coupling, that reflects changes in HbO and HbR measurements in the observed region (Ferrari and Quaresima, 2012; Scholkmann et al., 2014; Phillips et al., 2016). This can be interpreted as a change in tonic neural activity within that region (Owen et al., 2010). This activity can be measured with fMRI or electroencephalography (EEG), among other techniques. FMRI has high spatial and low temporal resolution, and it is expensive; on the other hand, EEG has low spatial and high temporal resolution. The advantages of fNIRS are its low cost, portability and possibility of use during daily activities, with a plausible spatial and temporal resolution (Nguyen and Hong, 2016; Hong and Zafar, 2018). The main disadvantage is that it does not evaluate infracortical layers, because light has a optimal penetration-scattering rate of 2 cm deep, suffering influence of the extracerebral superficial layers (Hoshi, 2016; Nguyen et al., 2016). Some authors postulate that fNIRS is a preferable tool to evaluate cortical activation induced by any type of electrical stimulation because it is less sensitive to electrical interference when compared to other neuroimaging techniques (Jang et al., 2014). fNIRS evaluating SSC can also be used to discriminate different stimulations, like handshake and cold temperature, as it presents different patterns of hemodynamic responses (Hong et al., 2017). Besides that, it is being used for the development of brain-computer interfaces (BCIs; Strait and Scheutz, 2014; Naseer and Hong, 2015), alone or together with others techniques as EEG (Khan et al., 2014; Hong and Khan, 2017).

Thus, to advance in the comprehension of the relationship between PES and the neural substrates at cortical areas involved in pain processing and understand possible therapeutic effects observed in clinical settings, this study assessed the changes on the concentration of HbO at DLPFC and SMC using fNIRS in healthy subjects that received accessory spinal nerve-peripheral electrical stimulation (ASN-PES). We tested the hypothesis that ASN-PES can promote cortical activation *via* bottom-up pathway on pain processing cortical areas modulated by top-down NIBS. Hence, this result can help to understand the clinical impact of PES on pain treatment and rehabilitation.

## Methods

The study protocol was approved by Hospital de Clínicas de Porto Alegre Ethics Committee Board (Institutional Review Board IRB 0000921), according to the Declaration of Helsinki. All subjects provided their written informed consent. The protocol was developed in accordance with the Consolidated Standards of Reporting Trials—CONSORT, and registered at ClinicalTrials.gov (NCT 03295370).

### Design Overview, Setting and Randomization

**Figure 1:**
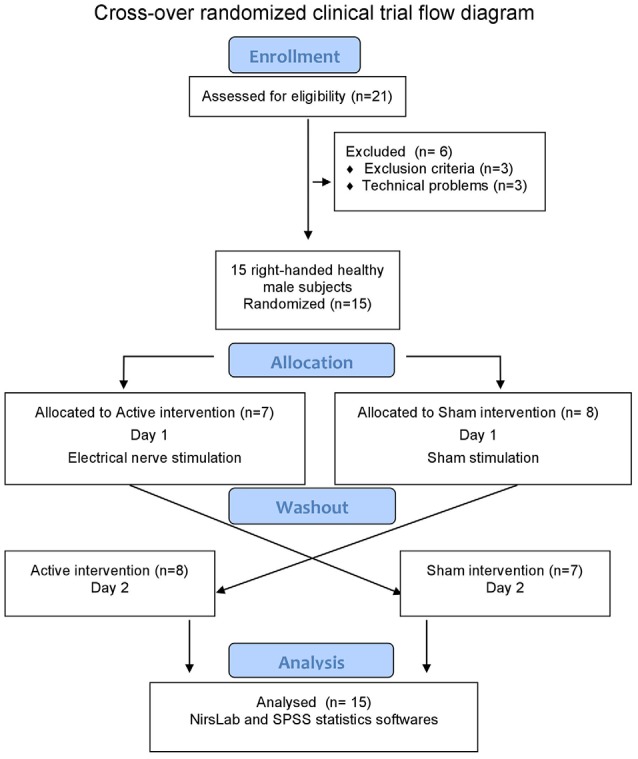
Flow diagram.

This crossover, sham-controlled clinical trial was carried out at Clinical Research Center of Hospital de Clínicas de Porto Alegre, Brazil. Healthy male volunteers, aged between 20 and 55 years, were recruited from the local community to undergo unilateral ASN-PES to evaluate cortical activation with fNIRS. Twenty-one right-handed, healthy male volunteers were eligible and agreed to participate. A standard screening questionnaire and a written consent was applied. Subjects could not have clinical co-morbidity, chronic pain, cerebral implants, history of neurologic or psychiatric disorders, BDI-II depression scale 12 or more and no drugs or alcohol abuse. Participants were instructed not to take analgesics, anti-inflammatory drugs, caffeine or any stimulant drinks at least 6 h prior to the intervention. The randomization plan to initiate the experiment in active or sham intervention was generated by specific software^1^. Six participants were excluded, three because of exclusion criteria application and three because they did not complete recording data due to technical problems with quality of signal on fNIRS calibration before starting the procedure. After a minimum interval of 6 days, participants were crossed-over to the second intervention. The study flow is represented in Figure 1.

For sample size estimation (minimum 12 subjects), we performed a internal pilot study with five subjects considering an effect size on changes on the concentration of HbO related to ASN stimulation equal to 0.8 for a standard deviation equal to 6.2 (error type II of 80% and error type I lower than 5%; Birkett and Day, 1994). The power of the initial estimative was confirmed at study end.

### Assessment of Demographic and Clinical Variables

Demographic data were assessed by a standard questionnaire. Beck II Depression Inventory (BDI-II) and Strait-Trait Anxiety Inventory (STAI) evaluated depressive and anxiety symptoms, respectively. The Pittsburgh Sleep Quality Index (PSQI) assessed sleep pattern.

### Assessment of Cortical Activation

**Figure 2:**
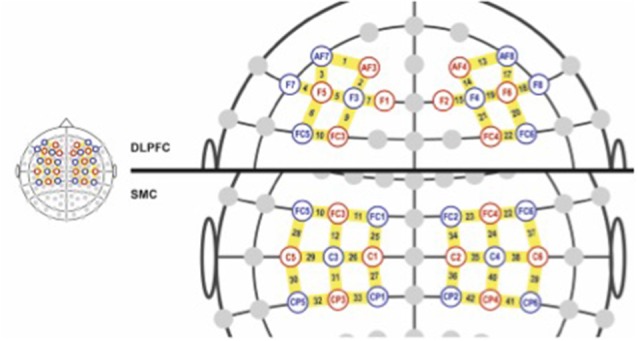
Cap montage. Sources (red), detectors (blue), in correspondence with 10/10 electroencephalography (EEG) system. Channels formed are in yellow bar. Dorsolateral prefrontal cortex (DLPFC) and sensorimotor cortex (SMC) areas are shown separately.

Cortical activation was assessed by fNIRS. We used a NIRx^®^ continuous waveform NIRScout 16 × 24 device, sampling rate of 3.91 Hz, dual-wavelength LED sources (760 nm and 850 nm), differential pathlength factor (DPF) of 7.25 for WL1 and 6.38 for WL2, for a distance between sources and detectors of 3 cm, as suggested by literature to evaluate cortical layers (Kohl et al., 1998; Zhao et al., 2002). Software equipment used was NIRStar 14.2 and nirsLAB 2017^2^. The montage intended to use as many channels (source-detector combination) as possible to cover motor and dorsolateral pre-frontal cortical bilateral areas, with a total of 40 measurement channels (Figure 2).

### Intervention

**Figure 3:**
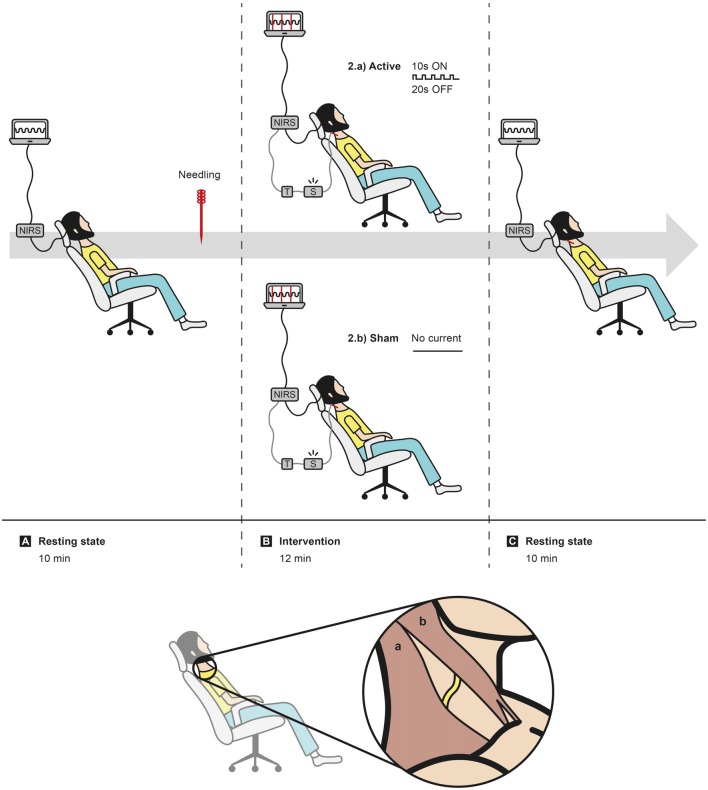
Experiment layout. Sequence of events: **(A)** 10 min of data acquisition in resting state, followed by needling right accessory spinal nerve (ASN); **(B)** randomization in active or sham intervention; **(C)** 10 min of data acquisition in resting state. In caption, representation of the subcutaneous location of the ASN (yellow trace) between trapezius muscle (a) and sternocleidomastoid muscle (b).

Subjects were seated on a comfortable reclining chair and asked to avoid any unnecessary movements. After the placement of the cap and software calibration checks, the signal was recorded for 10 min in resting state to surrounding accommodation. The right ASN was needled subcutaneously, at the right lateral cervical region, and the 0.25 × 40 mm sterilized acupuncture needle was fixed to the stimulator by a cable. A 12-min active or sham stimulation period was undertaken (720 s), followed by another 10 min resting-state period (Figure 3).

Electrical stimulation was undertaken with an EA stimulator (NKL 608^®^, made in Brazil) configured to apply a burst rectangular 200 μs-width current with maximum 5 mA of intensity on the needle. A special trigger marker device was developed to mark in registered data the exact moment the electrical current was discharged to the subject.

The active intervention consisted of 10 Hz electrical non-painful stimulus in burst current, 10 s ON and 20 s OFF, for 12 min, generating 24 blocks of hemodynamic curves in response to electrical current on unilateral right ASN. The intensity was determined during the first 2 min according to subject tolerance, in order to get mild or moderate muscular contraction of the right superior trapezius muscle for 10 s, followed by its relaxation for 20 s. In sham procedure, the intensity button was fixed on zero and there was no muscle contraction over the 12-min period, although it was previously provoked for the localization of ASN on needling phase. Thus, sham intervention had a very small electrical stimulation period (3–5 s).

A physician researcher with more than 10 years of needling experience conducted the study. The participants were not informed of intervention type on either day. At the end of each day of intervention, the subject filled a standard adverse effects questionnaire, adapted to the particularities of EA and fNIRS devices.

Based on Jurcak et al. (2007) and Koessler et al. (2009), validation of spatial resolution of scalp surface and its correlation with 10/10-system EEG parameters and Brodmann’s area, channels were grouped into four cortical areas of interest (CAI): left DLPFC, right DLPFC, left SMC and right SMC. Table 1 shows an approximate correlation of 10/10-system and cortical gyrus below, according to these authors. Note that the area called MOTOR includes sensory cortical zone, so it refers to SMC.

**Table 1:** Approximate anatomical correlation of international 10/10 EEG system and cortical gyrus (*n* = 40 channels).

**DLPFC**		
*10/10 system*	*10/10 system*	*Cortical lobe (gyrus)–Brodmann area*
AF3–AF7	AF4–AF8	Superior frontal BA 9–middle frontal BA 10
AF3–F3	AF4–F4	Superior frontal BA 9–middle frontal BA 8
F5–AF7	F6–AF8	Middle frontal BA 46–middle frontal BA 10
F5–F7	F6–F8	Middle frontal BA 46–inferior frontal BA 45
F5–F3	F6–F4	Middle frontal BA 46–middle frontal BA 8
F5–FC5	F6–FC6	Middle frontal BA 46–precentral frontal BA 6
F1–F3	F2–F4	Superior frontal BA 6–middle frontal BA 8
FC3–F3	FC4–F4	Middle frontal BA 6–middle frontal BA 8
FC3–FC5	FC4–FC6	Middle frontal BA 6–precentral frontal BA 6
**SMC**		
*10/10 system*	*10/10 system*	*Cortical lobe (gyrus)–Brodmann area*
FC3–FC5	FC4–FC6	Middle frontal BA 6–precentral frontal BA 6
FC3–FC1	FC4–FC2	Middle frontal BA 6–superior frontal BA 6
FC3–C3	FC4–C4	Middle frontal BA 6–postcentral parietal BA 123
C1–FC1	C2–FC2	Precentral frontal BA 4–superior frontal BA 6
C1–C3	C2–C4	Precentral frontal BA 4–postcentral parietal BA 123
C1–CP1	C2–CP2	Precentral frontal BA 4–postcentral parietal BA 7
C5–FC5	C6–FC6	Postcentral parietal BA 123–precentral frontal BA 6
C5–C3	C6–C4	Postcentral parietal BA 123–postcentral parietal BA 123
C5–CP5	C6–CP6	Postcentral parietal BA 123–supramarginal parietal BA 40
CP3–C3	CP4–C4	Inferior parietal BA 40–postcentral parietal BA 123
CP3–CP5	CP4–CP6	Inferior parietal BA 40–supramarginal parietal BA 40
CP3–CP1	CP4–CP2	Inferior parietal BA 40–postcentral parietal BA 7

*Adapted from Koessler et al. (2009)*.

### Data Processing and Statistical Analysis

While filtering and preparing the raw data, only the 12-min stimulation period was analyzed to observe the acute effects of electrical nerve stimulation on cortical hemodynamic response. Optical density changes recorded by the software was checked for quality and continuity; channels were considered adequate in a gain setting of 7 or less and coefficient of variation of 7.5% or less to improve the signal-to-noise ratio. To calculate HbO/HbR concentration changes using modified Beer-Lambert law, data were pre-processed with default band pass filters (low cut-off 0.01 Hz; high cut-off 0.2 Hz; Scholkmann et al., 2014). For each channel, the software computed the mean amplitude for hemodynamic response averaging the measurements of 10 s of stimulation from the baseline period, that is, before stimulus.

As fNIRS devices calculate the concentration changes of HbO/HbR in millimoles per liter (mmol/l or mM) in relative proportion related to a measured baseline, the synchronization of the electrical stimulation made by the trigger marker in recorded signals was essential to correct interpretation of data, since the peak of the standard hemodynamic response function (HRF) curve is 2–6 s from the stimulus onset. In our analysis, we used HbO relative concentration changes, since it is the most sensitive parameter of activity-dependent changes in optical measurements, compared to HbR and total hemoglobin (Tanosaki et al., 2001).

Data analysis was made by nirsLAB software by NIRx^®^ Technologies, using a general linear model (GLM) with the standard canonical HRF pattern, and statistical parametric mapping (SPM) Student’s *t*-test corrected for multiple comparisons, for the single subject level and for the group level. GLM coefficients were estimated by equation *Y = X*β *+ E*, where *Y* is the matrix of hemodynamic data; *X* is the design matrix; *β* is the GLM-coefficient matrix and *E* is the residual term. We used GLM parameters with no pre-whitening type of analysis, where the designed matrix used rest/stimulus to generate contrast 0/1 (nirsLAB 2017 manual^3^; Tak and Chul Ye, 2014).

Shapiro-Wilk test was used to evaluate normal distribution of the variables, and Student’s *t*-test was applied to evaluate differences between groups in parametric data. Multivariate analysis of covariance (MANCOVA) was used to assess statistical differences on multiple continuous dependent variables to verify differences regarding the activation of right and left DLPFC and right and left SMC areas. Comparisons were performed using a generalized estimating equation (GEE) model, followed by the Bonferroni correction for *post hoc* multiple comparisons. We analyzed the differences in HbO concentration changes by linear regression coefficients (Tak and Chul Ye, 2014), using SPSS version 22.0 (SPSS, Chicago, IL, USA). For all statistical analysis, the significance was set at *p* < 0.05.

## Results

Fifteen healthy right-handed male volunteers, mean 34.27 years old (±8.09), completed the 2-day study protocol. Demographic characteristics at baseline are shown in Table 2. No significant difference was found between groups that started with active or sham procedure on Day 1.

**Table 2:** Demographic characteristics between groups at baseline (*n* = 15).

	Active (*n* = 7)	Sham (*n* = 8)	*p*-value
Age (years)	36 (2.64)	32.75 (3.23)	0.458
Education (years)	19.43 (1.92)	19.5 (0.96)	0.973
Body Mass Index—BMI	26.6 (1.28)	24.1 (1.26)	0.186
Alcohol consumption	6/7	6/8	−
(≤1 week)			
Caffeine intake before	>6	>6	−
intervention (h)			
State-Anxiety Inventory (STAI)	22 (2.49)	19.75 (1.28)	0.420
Trait-Anxiety Inventory (STAI)	18.14 (1.45)	17.87 (1.29)	0.892
Beck Depression Inventory	4.86 (1.62)	2.25 (1.05)	0.190
(BDI-II)			
Pittsburgh Sleep Quality	4.29 (0.86)	3 (0.75)	0.281
Index (PSQI)			

*Comparisons using Student’s t-test for independent samples. Results are presented in mean and standard error*.

Minimal stress and/or mild muscular tension were reported before the experiment in some subjects (*n* = 5 in active and *n* = 8 in sham), without any major clinical manifestation. Four subjects complained of minimal to mild headache or cervical pain in both active and sham procedure, however, they were not able to distinguish if it was related to the fNIRS equipment (cap and optodes contact) or to the electrical stimulation *per se*. Prickling, itching, burning and/or heat sensation was mentioned by three subjects, related to the cap and optodes. The major discomfort mentioned was pain in the scalp, due to the tight cap and the pressure exerted by the optodes (*n* = 9 in active and *n* = 10 in sham). Somnolence was the most commonly reported symptom (24/30) in both active (*n* = 14) and sham (*n* = 10) procedures. The intensity of electrical current during active intervention required to get non-painful muscle contractions were minimal, as nerves need less electrical current to depolarize (1.133 mA ± 0.86). The electrical stimulation was well tolerated and asserted as non-painful by the participants. No relevant clinical complaint was observed.

We analyzed HbO concentration changes obtained in 30 experiments, 40 channels each, divided into active and sham group and into four CAI: left DLPFC, right DLPFC, left SMC and right SMC. The multiple dependent variables on MANCOVA model on CAI in active and sham groups are shown in Table 3. The effect of ASN electrical stimulation on HbO concentration changes was observed through the activation of right DLPFC (*F* = 5.572; *p* = 0.025) and left SMC (*F* = 4.542; *p* = 0.042) during the 10 s period of stimulation, compared to the 20 s period of rest, in active group but not in sham group. Regarding the activation of left DLPFC (*F* = 0.266; *p* = 0.610) and right SMC (*F* = 1.943; *p* = 0.174), there was no statistical difference between groups.

**Table 3:** Oxygenated Hemoglobin (HbO) concentration changes on Cortical Area of Interest (CAI) between groups (*n* = 15).

Dependent variable		Type III Sum of Squares	*df*	Mean Square	*F*	*p*	Partial Eta Squared
Left DLPFC		1.587 10^−9(a)^	1	1.587 10^−9^	0.266	0.610	0.009
Right DLPFC	Corrected Model	3.455 10^−8(b)^	1	3.455 10^−8^	5.572	0.025	0.166
Left SMC		5.001 10^−8(c)^	1	5.001 10^−8^	4.542	0.042	0.140
Right SMC		1.076 10^−8(d)^	1	1.076 10^−8^	1.943	0.174	0.065
Left DLPFC		2.018 10^−8^	1	2.018 10^−8^	3.382	0.077	0.108
Right DLPFC	Intercept	2.131 10^−8^	1	2.131 10^−8^	3.437	0.074	0.109
Left SMC		2.510 10^−8^	1	2.510 10^−8^	2.280	0.142	0.075
Right SMC		3.190 10^−8^	1	3.190 10^−8^	5.763	0.023	0.171

*Univariate Tests: F tests the effect based on linearly independent pairwise comparisons among the estimated marginal means. Test of Between-Subjects Effects; Multivariate Tests Observed Power = 1, 0. ^(a)^R Squared = 0.009 (Adjusted R Squared = −0.026); ^(b)^R Squared = 0.166 (Adjusted R Squared = 0.136); ^(c)^R Squared = 0.140 (Adjusted R Squared = 0.109); ^(d)^R Squared = 0.065 (Adjusted R Squared = 0.031)*.

**Figure 4:**
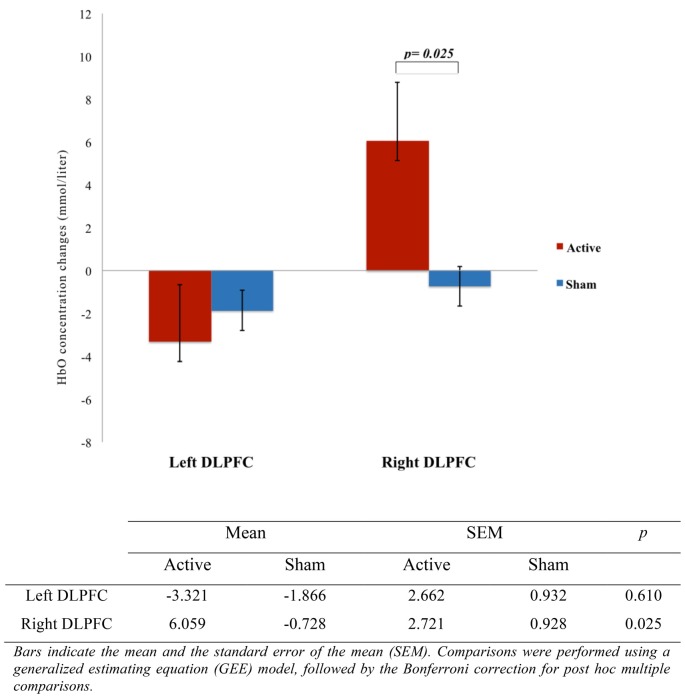
Comparison of DLPFC activation between active and sham groups (*n* = 15). The figure shows a representation of the mean oxygenated hemoglobin (HbO) concentration changes, measured in millimoles per liter (mmol/l) with correspondent *p*-value, indicating the difference of right DLPFC activation during accessory spinal nerve-peripheral electrical stimulation (ASN-PES).

**Figure 5:**
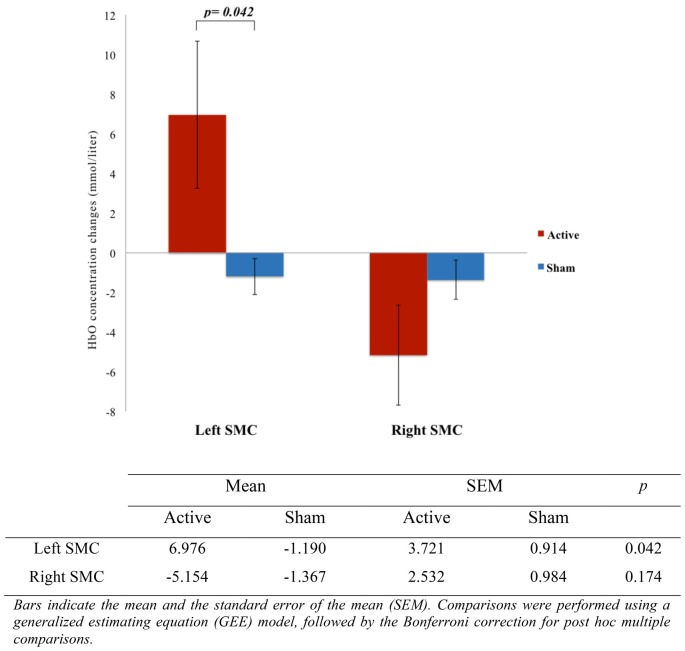
Comparison of SMC activation between active and sham groups (*n* = 15). The figure shows a representation of the mean HbO concentration changes, measured in millimoles per liter (mmol/l) with correspondent *p*-value, indicating the difference of left SMC activation during accessory spinal nerve-peripheral electrical stimulation (ASN-PES).

**Figure 6:**
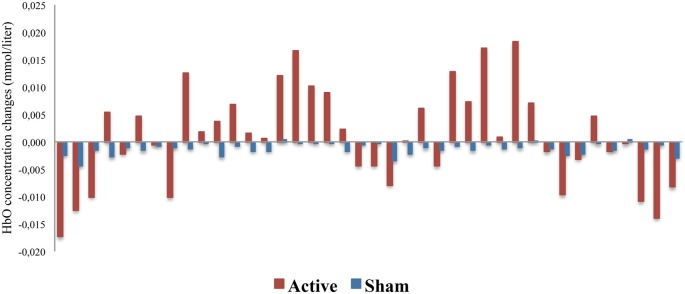
HbO concentration changes in each channel between active and sham groups (*n* = 40). The figure shows a representation of the mean HbO concentration changes, measured in millimoles per liter (mmol/l), in each channel, demonstrating subtle changes in oxy-hemoglobin between active and sham groups in almost all of 40 channels.

**Figure 7:**
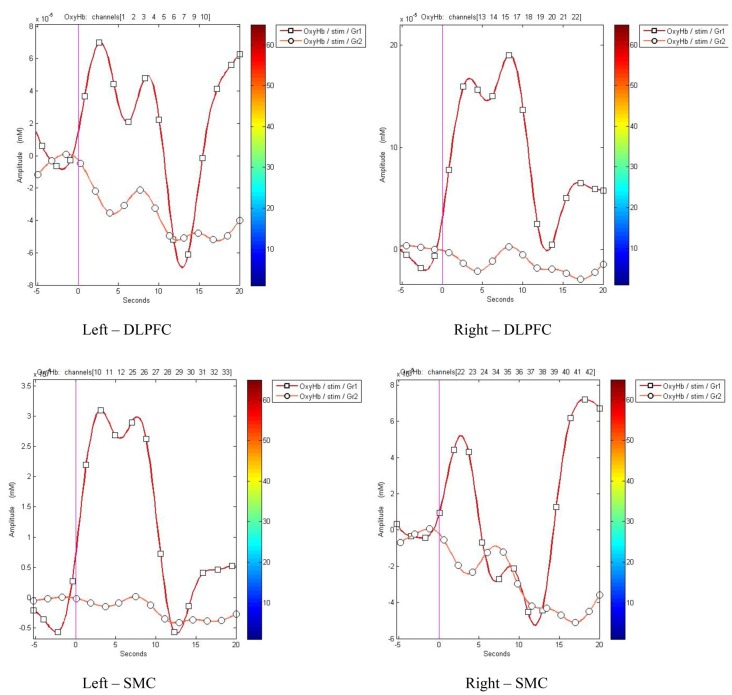
HbO mean curves for each cortical area of interest between groups. Channels were gathered to display the oxy-hemoglobin changes for active (Gr1) and sham (Gr2) groups, from 5 s before (baseline) to 20 s after stimulation onset.

**Figure 8:**
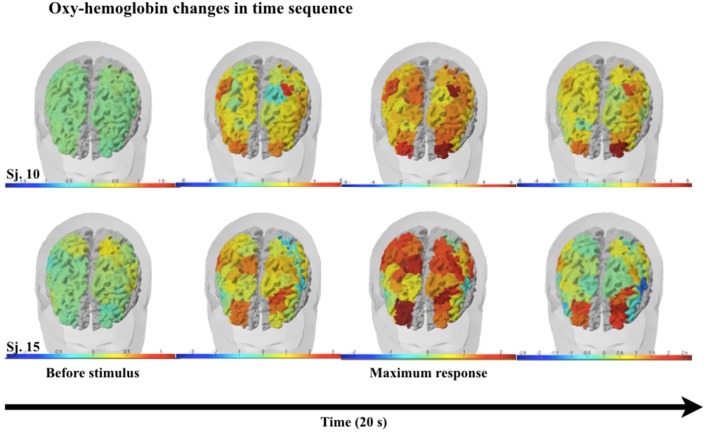
Graphic representation frames on a 3D brain surface model in two subjects, showing oxy-hemoglobin changes in time sequence. Color bars represent activation (red) or deactivation (blue) response. The first frame represents oxy-hemoglobin before the electrical stimulus, followed by frames in time sequence.

The representation of DLPFC and SMC activation between active and sham groups during ASN-PES are showed in Figures 4, 5, respectively, with mean HbO concentration changes in millimoles per liter (mmol/l), standard error of the mean (SEM) and correspondent *p*-value. Figures 6–8 show different representations of the same results found in statistical analysis. Additional data from each channel are available at Supplementary Material section. In HbO mean curves for each cortical area of interest shown in Figure 7, note that the 10 s stimulation time has a different pattern than the subsequent rest period.

## Discussion

This study confirms our hypothesis that the ASN-PES can promote cortical activation on areas involved in pain and emotion, nominally SMC and DLPFC. Our findings showed that unilateral right ASN electrical burst stimulation with 10 Hz 10 s ON and 20 s OFF was able to activate ipsilateral dorsolateral prefrontal (DLPFC) and contralateral sensorimotor (SMC) cortical areas during stimulation. ASN-PES induced changes in regional cerebral blood flow in central pain-related regions, significantly increasing the perfusion in those areas in active but not in sham stimulation. Thus, it was able to produce bottom-up activation to central brain regions of pain processing.

The relevance of these results is to extend literature upon PES effects to modulate cortical areas involved in pain processing and help to investigate neurobiological mechanisms of peripheral neuromodulatory techniques. Furthermore, it helps to understand systemic effects observed in clinical practice and supports the possibility of using this type of non-painful peripheral stimulation as a therapeutic approach in pain treatment, including the possibility to use combined methods to induce a top-down (e.g., NIBS or behavioral therapies) and bottom-up modulation (e.g., dry-needling). It also allows more understanding on pain mechanisms considering its dimensions, which comprises sensory-discriminative, affective-motivational and cognitive-behavioral aspects (Melzack, 2001), as these manifestations are linked to neural networks of SMC and DLPFC.

Regarding the activation of SMC, our work is lined up to previous results in the literature and suggest that the temporal resolution of fNIRS offers an efficient technical solution to study the cortical areas activated by PES. However, in left DLPFC and right SMC, we did not find statistical difference between baseline and 10 s stimulation, but we observe that there is a subtle rise in HbO concentration towards activation on subsequent 10 s of rest, as shown in Figure 7. Although it can be associated to an error type, another hypothesis is that these areas are also activated, with a temporal delay, in active but not in sham group. Another hypothesis is that some targets areas are activated in detriment of deactivation of others. Indeed, temporal changes were found by others authors, as decrease of cortical activation during execution of hand movements using fNIRS after 5 min of electrical stimulation (Jang et al., 2014). Besides that, parts of activated circuits and subsequent temporal responses seem to be enrolled by inter-hemispheric functional connections (Sankarasubramanian et al., 2017). Furthermore, different functions of the right and left hemispheres, right and left DLPFC and medial and lateral PFC sub-regions in pain processing and in unpleasant sensations are involved in neural networks not yet clarified (Lorenz et al., 2003; Cieslik et al., 2013; Brasil-Neto, 2016).

This study added value to the fact that ASN-PES is **non-painful** and utilize intensities above motor threshold. The goal of ASN needling is not to cause pain in the subcutaneous insertion of the needle in the cervical region, tangentiating the nerve to get its depolarization. The electrical current must flow through the perinervous layer, without hurting the nerve tissue. This causes mild to moderate movement of the muscles under ASN domain, i.e., trapezius muscle and sometimes sternocleidomastoid muscle, without pain. It has the same goal as functional electrical stimulation (FES), where a non-painful electrode stimulus generates action potentials resulting in contraction of the target muscles. In Blickenstorfer et al.’s (2009) study with FES, fMRI showed activation pattern in the contralateral M1, S1, PMC and the ipsilateral cerebellum, as well as bilateral S2, SMA and ACC.

It is conceivable that the bottom-up activation of DLPFC induced by ASN-PES may trigger top-down responses, since ACC is implicated in the elicitation and control of sympathetic autonomic arousal. Therefore, the activation of right DLPFC by ASN may culminate in nucleous accumbens (NAc) activation in order to activate pain descending modulatory system together with PAG and rostroventral medulla (RVM; Navratilova and Porreca, 2014; Elman and Borsook, 2016). This pathway could explain the sense of relaxation and well being reported by subjects following the active intervention.

We observed that stimulation of right ASN produced similar results seen during VN stimulation with electrodes and implanted devices (Frangos and Komisaruk, 2017). During that study, fMRI images showed ipsilateral activation of nucleus of solitary tract (NST), which is the primary central relay of vagal afferents, insula, thalamus, caudate nucleus and SSC; deactivation occurred in hippocampus, contralateral NST and ipsilateral spinal TN. In a subsequent period, activation was observed in substantia nigra, ventral tegmental area (VTA), dorsal raphe nuclei (DRN) and PAG. Based on our findings, we cannot affirm that the ASN-PES involves the activation of subcortical areas, but the anatomical correlation of ASN and VN raises an intriguing question to be explored in future studies. The anatomical structure of ASN gives us biological support to investigate the ASN-PES as a more accessible alternative for routine clinical use when therapeutic approach is to target the VN.

Also, a better comprehension of ASN-PES effect as a bottom-up neuromodulatory approach is its potential to be combined with other top-down NIBS techniques, such as transcranial direct current stimulation (tDCS) and TMS. The argument to support this question is a potential additive effect and, consequently, a better clinical response. A previous study that applied tDCS together with PES over the median nerve found increase in MEP compared to baseline in TMS parameters (Rizzo et al., 2014). Other study showed frequency-dependent motor cortex response with combined TMS and PES to test bi-directional plasticity (Pitcher et al., 2003). Combined PES and tDCS intervention on patients with chronic low back pain improved symptoms than either intervention alone or sham in another trial (Schabrun et al., 2014). In addition, a systematic review on stimulus parameters of PES in healthy subjects demonstrated that higher intensities of stimulation produced more consistent effects on the increase in excitability of the corticomotor pathway (Chipchase et al., 2011a). In another study, IMS [which appears to encompass the same type of stimulus as EA (Kim et al., 2012)] enhanced inhibitory modulation in cortical and infracortical pain processing systems when applied to women with knee osteoarthritis undergoing tDCS (Tarragó et al., 2016). Possibly, modulatory techniques such as NIBS and PES attempt to re-reorganize neural circuits, improving malfunction of the whole system on cortical, infracortical, spinal and local sites.

### Study Limitations

It is necessary to point out some limitations concerning this study. We did not have a 3D device to confirm the probe location to relate it to Brodmann’s areas. Instead, we used the 10/10 International System, as shown in Table 1. Likewise, we did not have short distance inter-probes, which would have helped to control noise data from skin blood flow, although we did not place probes in the forehead (Takahashi et al., 2011). Scalp hemodynamics often contaminates fNIRS signals, and standard source-detector distance channels tend to over-estimate the artifacts (Sato et al., 2016). These limitations interfere with the evaluation of cortical activation. Actually, fNIRS technical limitations include superficial depth cortical evaluation, cardiovascular frequency noise, environmental light noise and motion artifacts (Ferrari and Quaresima, 2012; Scholkmann et al., 2014; Tak and Chul Ye, 2014). Furthermore, as it was already pointed out, a single-session of unilateral electrical stimulation of a craniocervical nerve can tell us about its acute manifestations without temporal changes, that can be different in subsequent measurements, as pointed out by other authors (Tanosaki et al., 2001; Jang et al., 2014; Frangos and Komisaruk, 2017).

While a physiological basis study on cortical responses, we must consider the amostral design that included only right ASN stimulation in healthy, right-handed males in a controlled environment. As expected, we observed large inter-individual responses, which might be due to a particular cortical organization or anatomical features, such as skull and subcutaneous tissues thickness, head format and skin or hair pigmentation (Niederhauser et al., 2008). Variables such as tiredness, stress, muscular tension, anxiety, expectancy, fear of pain, discomfort due to sitting still or cap pressure can change mental status, which can activate unexpected areas; this may be the reason why sham procedure data showed more variability than active stimulation data. Females were not included in our study to avoid hormonal influences on results, as women are more susceptible to negative emotional responses such as fear of pain, stress and anxiety (da Silva et al., 2015). The exclusion of females may generate either better or worse cortical responses to stimulation. Response patterns may also be different with bilateral stimulation in healthy vs. chronic pain patients. Other variables, such as age, lifestyle, education level, genetics and even recent news about chronobiology may play a fundamental role on response patterns in other subgroups that experience top-down or bottom-up modulations (Cummings and Baldry, 2007; Ridding and Ziemann, 2010).

Moreover, we observed that studies related to PES are very heterogeneous with unstandardized nomenclature, protocols, electrical features, duration and type of stimulus (Chipchase et al., 2011a; Rossini et al., 2015; Chakravarthy et al., 2016). It is necessary to develop an academic consensus aiming to standardize research and clinical protocols since PES techniques seem to be a promising therapeutic tool for pain management and neuro-rehabilitation.

### Conclusions

In conclusion, cortical activation of sensorimotor and DLPFC induced by non-painful ASN-PES seems to activate the same crucial pain cortical related areas, acting on bottom-up modulation pathway. Also, it opens a novel window of research into the possibilities of ASN-PES on modulation for treatment purposes. Further studies are needed in order to explore this technique as a potential therapeutic tool and its impact in clinical settings.

## Ethics Statement

The study protocol was approved by Hospital de Clínicas de Porto Alegre Ethics Committee Board (Institutional Review Board IRB 0000921), according to the Declaration of Helsinki. All subjects provided their written informed consent. The protocol was developed in accordance with the Consolidated Standards of Reporting Trials—CONSORT, and registered at ClinicalTrials.gov (NCT 03295370).

## Author Contributions

All authors made a significant contribution to: (a) the study concept and design, acquisition of data, or analysis and interpretation of data; (b) drafting/revising the manuscript for important intellectual content; and (c) approval of the final version to be published.

## Conflict of Interest Statement

The authors declare that the research was conducted in the absence of any commercial or financial relationships that could be construed as a potential conflict of interest.

## Footnotes

^1^www.randomization.com

^2^www.nirx.net

^3^www.nirx.net

## Abbreviations

ACC: Anterior Cingulate Cortex
ASN: Accessory Spinal Nerve
CA: Cortical Activation
CAI: Cortical Area of Interest
DLPFC: Dorsolateral Prefrontal Cortex
EA: Electroacupuncture
fNIRS: Functional Near Infrared Spectroscopy
fMRI: Functional Magnetic Ressonance Imaging
HbO: Oxygenated Hemoglobin
HbR: Deoxygenated Hemoglobin
HRF: Hemodynamic Response Function
IMS: Intramuscular Stimulation
M1: Primary Motor Cortex
MC: Motor Cortex
NIBS: Non-invasive Brain Stimulation
NMES: Neuromuscular Electrical Stimulation
PAG: Periaqueductal Gray
PES: Peripheral Electrical Stimulation
PFC: Prefrontal Cortex
PMC: Premotor Cortex
S1/SI: Primary Somatosensory Cortex
S2/SII: Secondary Somatosensory Cortex
SMA: Supplementary Motor Area
SMC: Sensorimotor Cortex
SSC: Somatosensory Cortex
VN: Vagus Nerve.
