# Supplementary figures and images for: Functional Spectroscopy Mapping of Pain Processing Cortical Areas During Non-painful Peripheral Electrical Stimulation of the Accessory Spinal Nerve
Source: Front Hum Neurosci. 2019 Jun 13;13:200. doi: 10.3389/fnhum.2019.00200 (PMC6585570; doi:10.3389/fnhum.2019.00200)

1 **7. Appendices**

2 **Supplementary data - HbO mean curves in each channel between groups (40 channels).**

3  
4

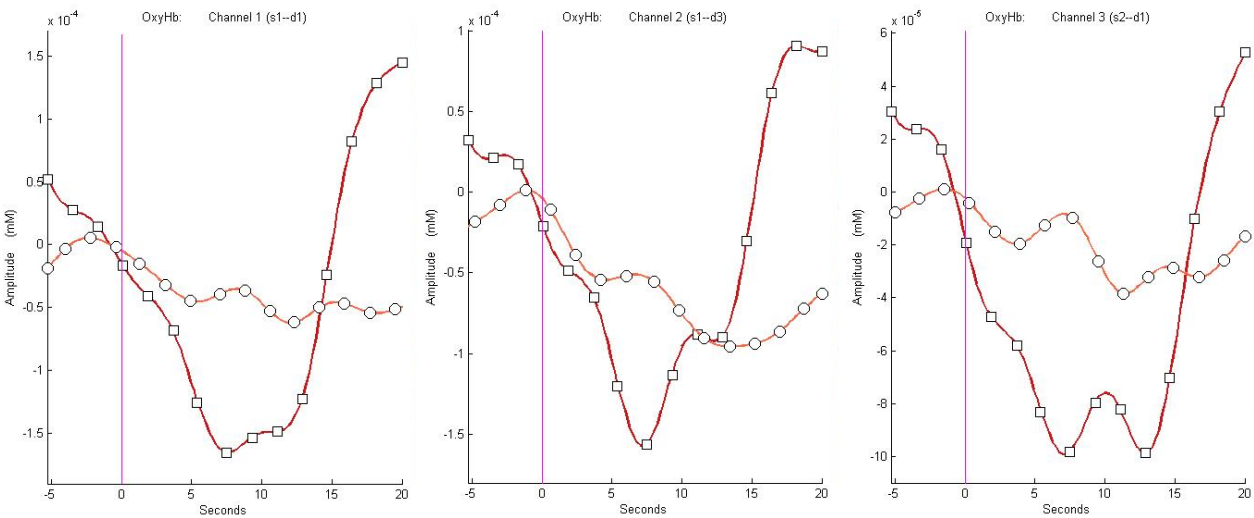

5

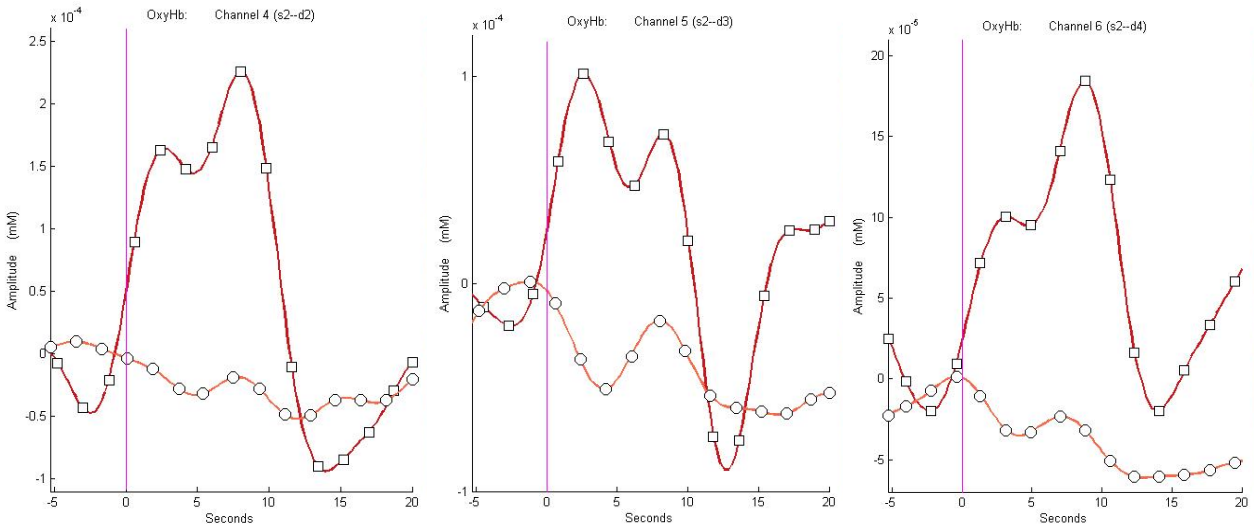

6  
7

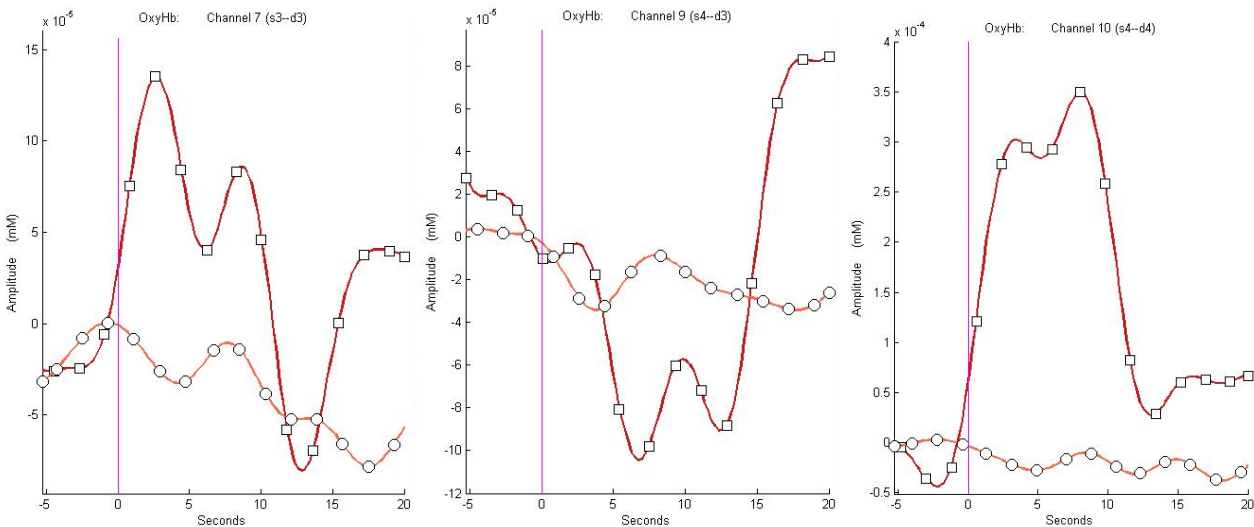

8

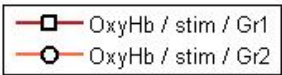

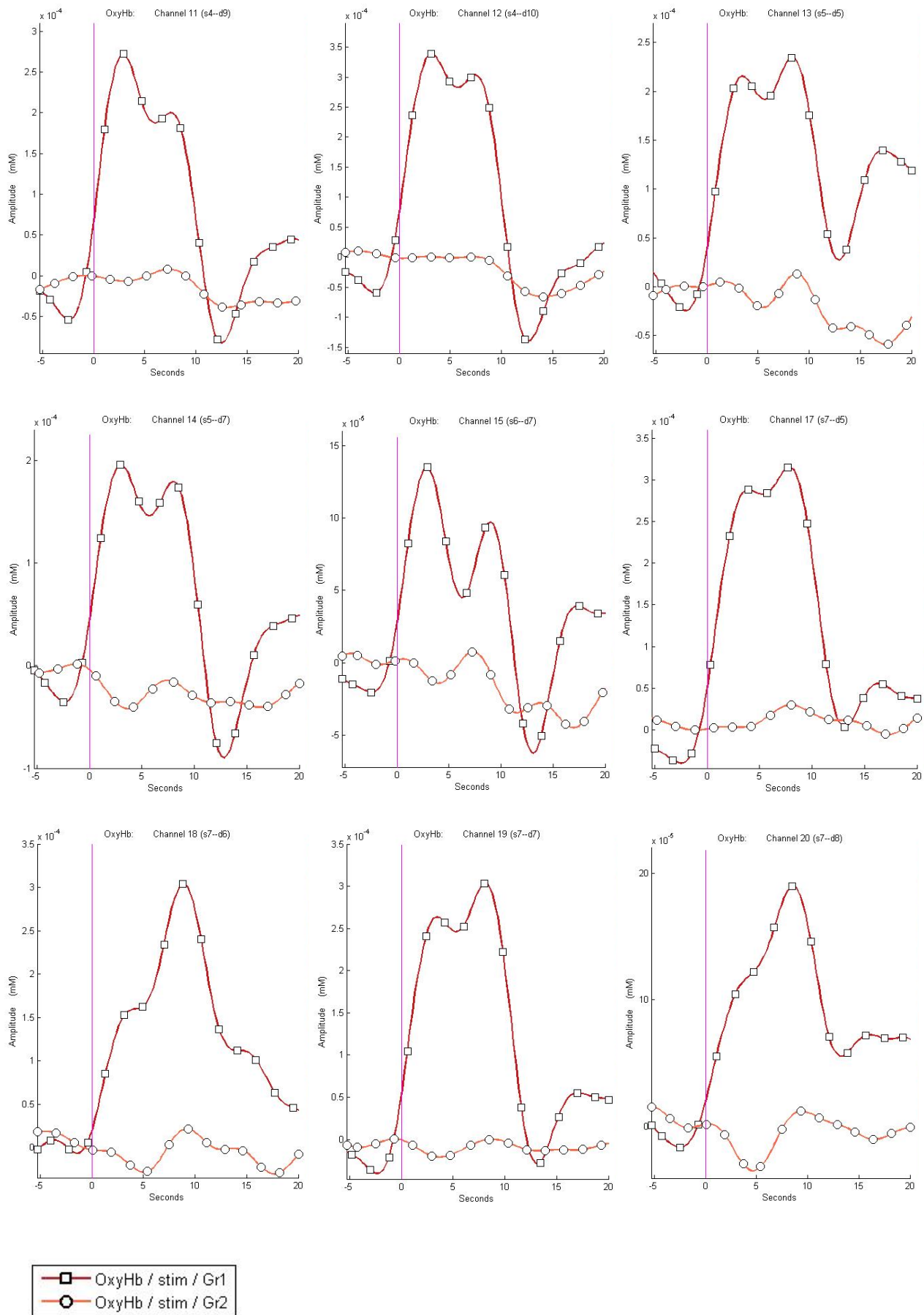

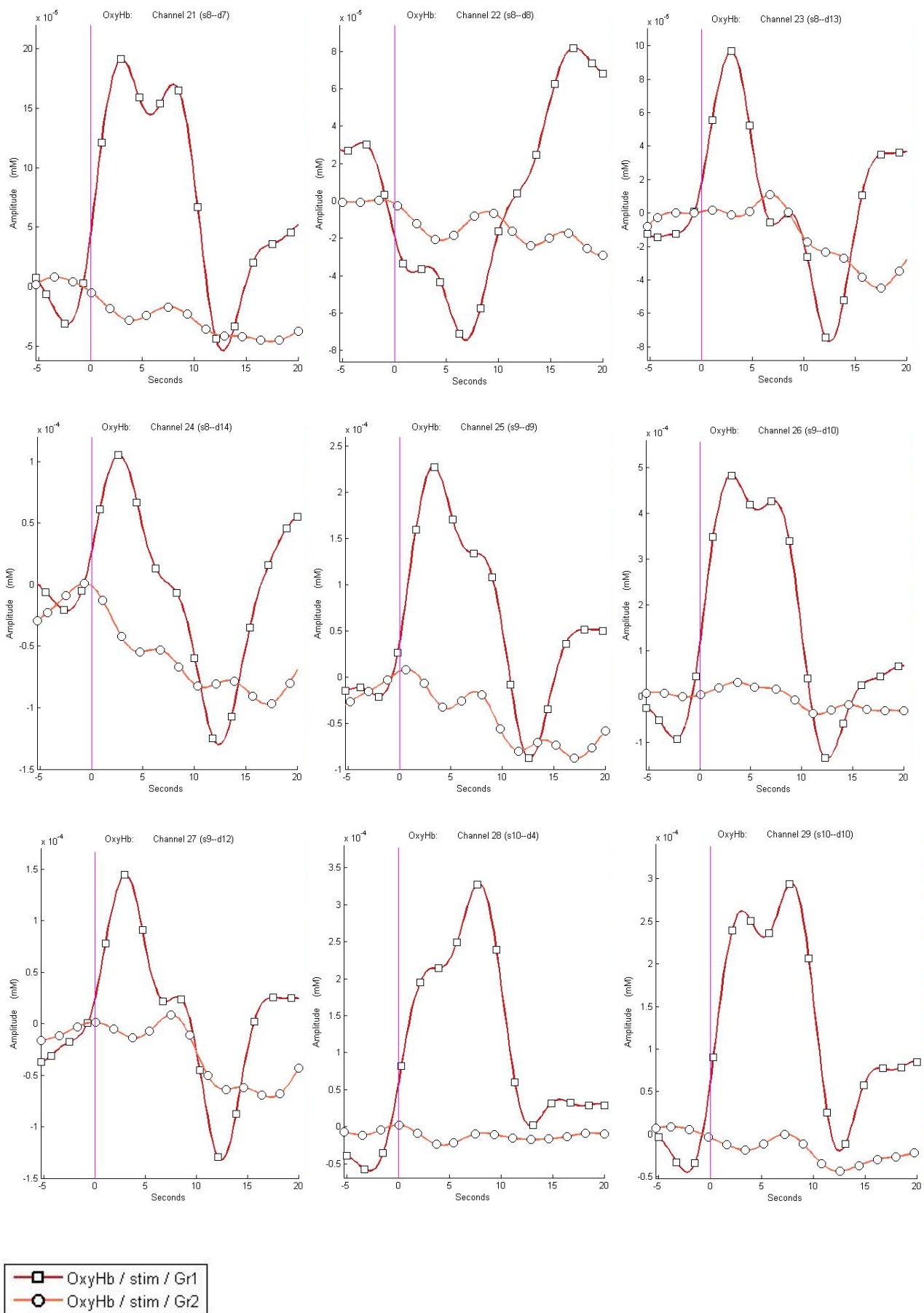

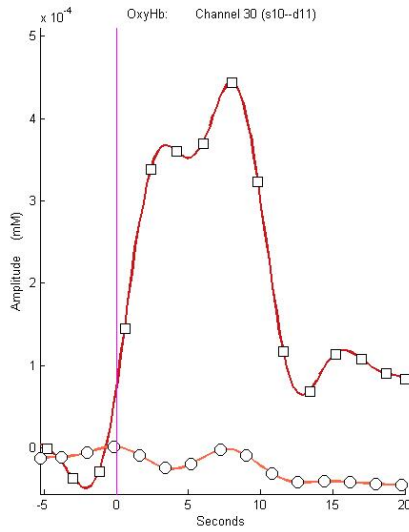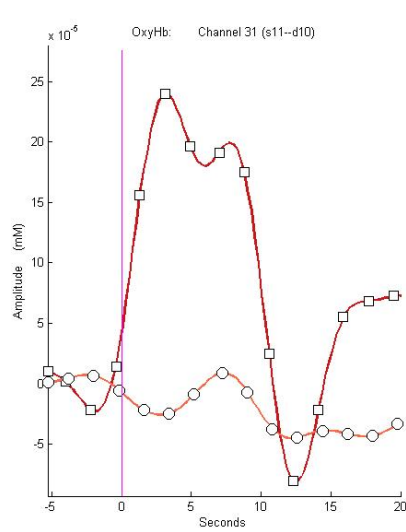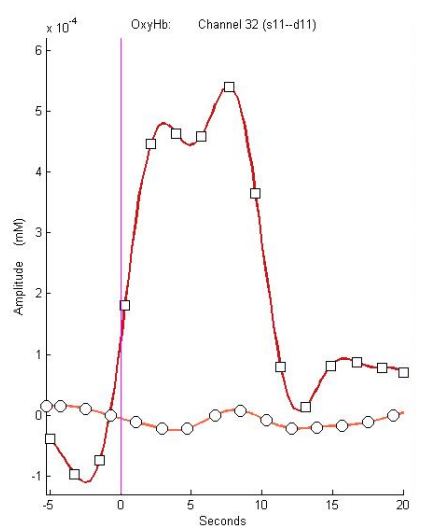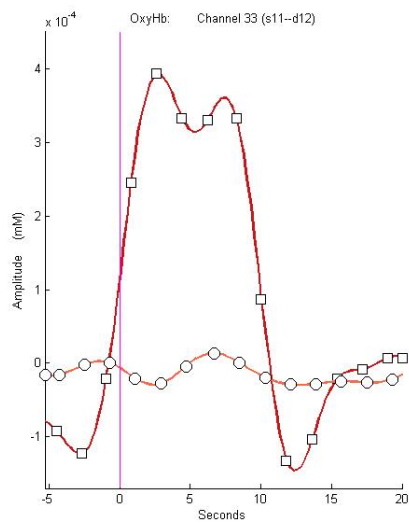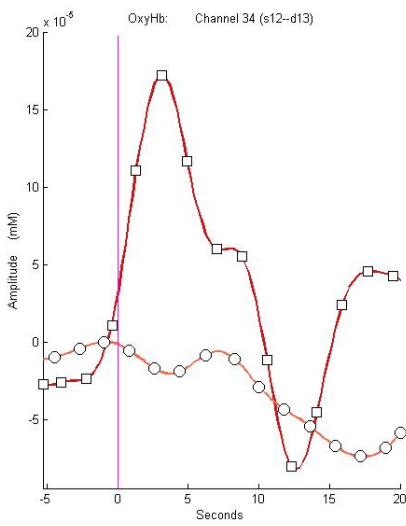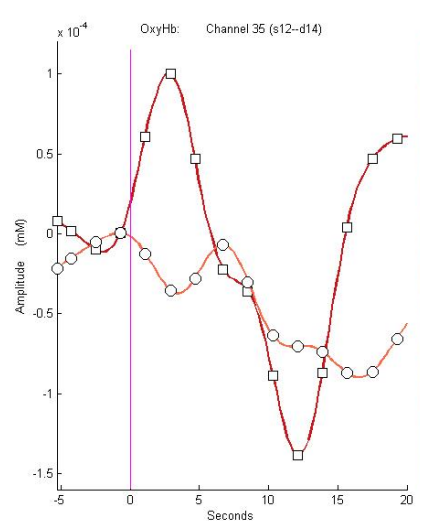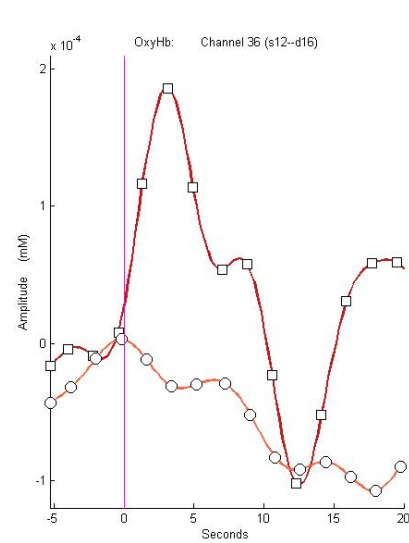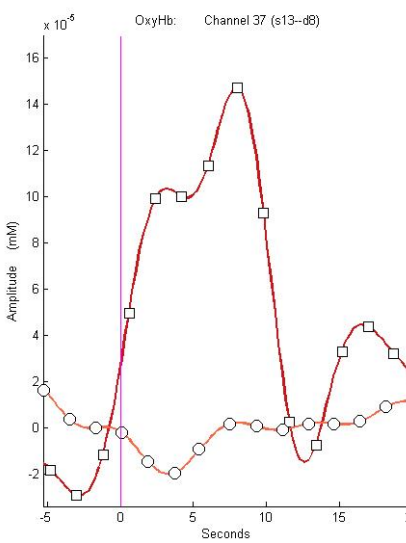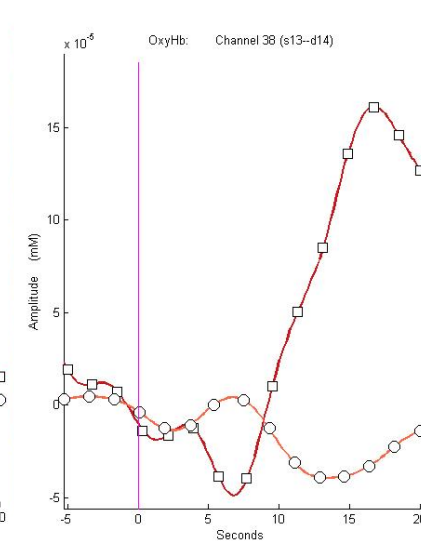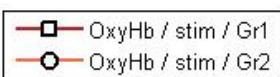

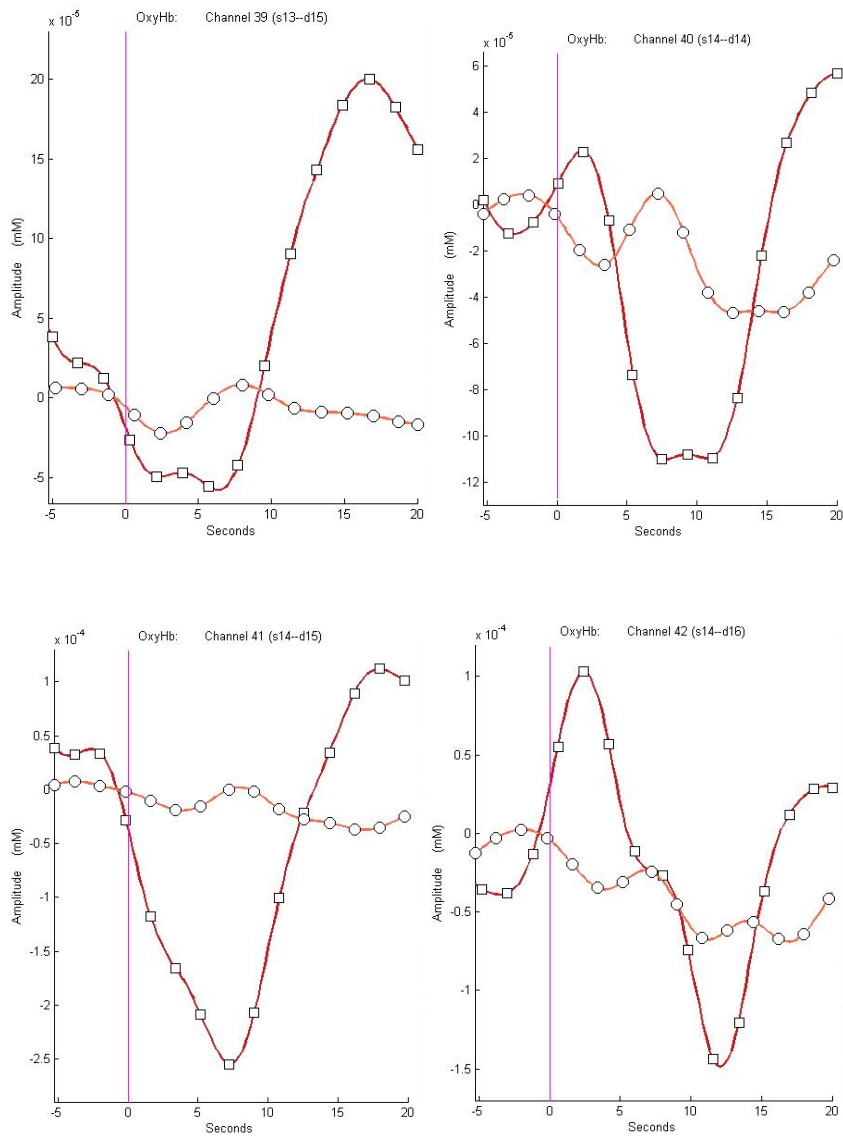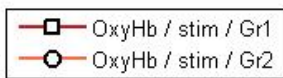

Supplement: Supplementary file 1 [file Data_Sheet_1.pdf]
